# Characteristics of primary exertional headache in Korean marine corps

**DOI:** 10.1186/1129-2377-14-S1-P39

**Published:** 2013-02-21

**Authors:** BH Cho, YUN Choi, TS Nam, SM Choi, SH Lee, MS Park, MK Kim

**Affiliations:** 1 Department of Neurology, Chonnam National University Medical School, Gwangju, Korea, Republic of; 2 Department of Neurology, Chonnam National University Hwasun Hospital, Hwasun, Jeonnam, Korea, Republic of

## Introduction

Primary exertional headache is included in "Other Primary Headaches" (Group 4) in the International Classification of Headache Disorders, 2nd edition (ICHD-II) with primary cough headache, primary sexual headache, and idiopathic stabbing headache. The prevalence of primary exertional headache showed about 1-30.4%.

## Purpose

We investigated about prevalence and characteristics of primary exertional headache in Korean marine corps.

## Methods

704 patients were treated due to headache. 79 patients of them suffered from exertional headache. We assessed characteristics of patients, comorbidity of migraine, nature of headache, Visual Analog Scale(VAS), neurologic symptoms, exercise which prompted the headache, and effect of medication. Effect of medication also evaluated with VAS.

## Results

10.38% of headache group was diagnosed as primary exertional headache. Average age was 20.6 year old and only 1 of 73 patients was female because of homogeneity of military corps. Most quality of headache is pulsating nature (93%) and most location of the headache is bilateral(84%). 3 patients had history or comorbidity of migraine(4%). 16 patients had accompanied symptoms, such as nausea(13 patients), photophobia(1 patient), dizzy sense(2 patients). In analyses of provoked factor, anaerobic exercise is most prompted cause of the headache (67%). 15 patients were provoked due to swimming, and other patients suffered from headache after running, or other aerobic exercise. Average of VAS score was 8.48. After treatment, the score was decreased to 3.40.

## Conclusion

Prevalence of primary exertional headache in Korean marine corps is similar to previous population-based studies. Low rate of comorbidity of migraine and neurologic symptoms was differed from these studies. Anaerobic exercise and swimming are revealed frequent inducing factors of primary exertional headache. Because of significant lowering VAS scale, ergotamine might be useful drug to treat the headache.
